# Changes in retirement plans in the English older population during the COVID-19 pandemic: The roles of health factors and financial insecurity

**DOI:** 10.1007/s10433-023-00770-1

**Published:** 2023-06-13

**Authors:** Claryn S. J. Kung, Jingmin Zhu, Paola Zaninotto, Andrew Steptoe

**Affiliations:** University College London, London, UK

**Keywords:** COVID-19 pandemic, English Longitudinal Study of Ageing, Retirement planning, Mental health, Self-rated health, Financial insecurity

## Abstract

Over the course of 2020 and 2021, the COVID-19 pandemic disrupted lives globally. In the UK, unemployment rate continued to increase during and post-lockdown periods, and job security and financial wellbeing deteriorated. It is important to understand whether individual decisions related to retirement plans have changed systematically as a result of the pandemic, especially among older adults who experienced greater rates of pandemic unemployment. Using the English Longitudinal Study of Ageing, this article examines changes in retirement plans of older adults during the COVID-19 pandemic and estimates the impact of health and financial circumstances on these changes. In June/July 2020, 5% of 2095 participants reported planning earlier retirement, while 9% reported planning later retirement. We found that poor self-rated health and financial insecurity were associated with intentions to postpone retirement. Additional risk of later retirement associated with poor health was detected among those experiencing financial insecurity. In November/December 2020, 7% of 1845 participants reported planning earlier retirement, while 12% reported planning later retirement. We found that poor health was predictive of a lower relative risk of later retirement, while depressive symptomology and financial insecurity predicted a higher relative risk of later retirement. The findings imply a contextual role of health factors in, and a persistent influence of financial insecurity on, retirement planning in the older population.

**Supplementary Information:**

The online version contains supplementary material available at 10.1007/s10433-023-00770-1.

## Introduction

Over the course of 2020 and 2021, the COVID-19 pandemic disrupted lives globally, with fear and uncertainties surrounding the novel coronavirus and its mutations, lockdown restrictions and social distancing policies. Older adults constituted a vulnerable population during this period, that is, they were more likely to develop serious conditions and experience higher mortality if infected—many were required to shield or stay at home, leading to social isolation and poor mental health (Di Gessa and Price 2022). It was reported that as the unemployment rate kept increasing during and post-lockdown periods, job security and financial wellbeing deteriorated in the UK (Cheng et al. 2021; Brown et al. 2022). What has been less discussed, however, is the impact of the pandemic on older workers, even though there is evidence for greater rates of pandemic unemployment among older than among younger workers (Bui et al. 2020). There was also a depression of pension values due to the market downturn, which would have affected older workers close to retirement age (Sutcliffe 2020; Pew Charitable Trusts 2021). Those financially affected by the pandemic have also been shown to be less likely to save and annuitise (Hurwitz et al. 2021).

From a policy and planning perspective, it is important to understand whether individual decisions related to retirement plans have changed systematically as a result of the pandemic. The literature has shown that retirement plans are significantly influenced by personal (including health) and financial factors (Quinn 1977; Taylor and Shore 1995; Delpachitra and Beal 2002; Scharn et al. 2018), both of which have been substantially altered by the pandemic. Moreover, the pandemic presented unique challenges compared with past recessions: for instance, working longer to compensate for the decline in retirement savings may have been less viable, given older adults’ need to shield or stay at home (Bui et al. 2020). Ageism may have also been a greater problem than it was previously, considering the increasing need for digital skills and reliance on remote working (Pit et al. 2021). However, few studies have documented whether and how older workers have changed their retirement plans during this crisis and the impact of health and financial circumstances on these changes (Davis 2021; Kaur 2021). Moreover, the intersectionality between health factors and financial circumstances has not yet been revealed in the context of the pandemic.

Using the English Longitudinal Study of Ageing (ELSA), a nationally representative study on older adults aged 52 years and above in private households in England, evidence is provided on how health factors and financial insecurity during the COVID-19 pandemic have affected retirement plans. Older adults were observed at two peak time points of the pandemic—June/July 2020 and November/December 2020, with a 74% response rate—thereby allowing an examination of whether the impact of these factors remained stable over time, and a longitudinal analysis that accounts for individual unobserved heterogeneity. In addition, interaction effects of health factors and financial insecurity on changes in retirement plans are investigated in this article.

## Theoretical framework

In studying factors influencing retirement plans or decisions, the push–pull theory, which distinguishes between ‘push’ and ‘pull’ routes of influence on retirement, is one of the most important and widely discussed theories. People are ‘pushed’ to quit their job due to negative factors that constrain them from working (Shultz et al. 1998; Oksanen and Virtanen 2012; De Preter et al. 2013), such as poor health, poor working conditions, caring responsibilities, and so on (Olesen et al. 2012; Oakman and Wells 2013; Qvist 2021). They can also be ‘pulled’ towards retirement (Oksanen and Virtanen 2012), due to positive factors encouraging earlier retirement, including leisure expectations and pension wealth, among others (Blöndal and Scarpetta 1999; Munnell et al. 2004).

Health factors have been widely investigated as predictors of retirement (Mein et al. 2000; Munnell et al. 2004; Topa et al. 2009; Olesen et al. 2012; Scharn et al. 2018). Poor physical and mental health are usually considered push factors of retirement behaviours and plans (Taylor and Shore 1995; Von Bonsdorff et al. 2010; Olesen et al. 2012). Theoretically, poor health affects both people’s ability and desire to work. People with poor physical or mental health may experience a loss of control over their work (Topa et al. 2009), affecting their productivity and ability to do full-time work, thus forcing them into retirement. Moreover, restricted ability to work promotes people’s self-efficacy of retirement—their self-perceived capability to carry out retirement successfully (Hoffmann and Plotkina 2021)—which contributes to retirement intentions (Taylor and Shore 1995). Meanwhile, a positive attitude and orientation towards leisure activities drive people to quit the labour market, as suggested by Beehr’s model (Beehr 1986). Therefore, hypotheses 1 and 2 are constructed accordingly:

- Hypothesis 1. People in poor health are more likely to retire earlier, or less likely to retire later.
- Hypothesis 2. People with psychological distress are more likely to retire earlier, or less likely to retire later.

The impact of financial insecurity on retirement plans is less straightforward. On the one hand, financial security acts as a pull factor of earlier retirement. A higher level of financial security is associated with earlier retirement (Taylor and Shore 1995), which conversely implies that financial insecurity or stress tends to retain people in employment (Mein et al. 2000). On the other hand, financial insecurity has a detrimental influence on health factors. Studies have shown that during the COVID-19 pandemic, financial insecurity or concerns led to the deterioration of mental health worldwide (Wilson et al. 2020; Cheng et al. 2021; De Miquel et al. 2022). Following an adaptation of the model proposed by Homaie Rad et al. (2017), there exists a trade-off between the direct effect of financial insecurity on retirement and the indirect effect via health factors. When the positive utility of financial security is larger than the negative utility of poor physical or mental health, later retirement plans may be made. In the context of the COVID-19 pandemic, it is conjectured that people’s perceptions of their financial situation may be more volatile and influential on retirement planning than the changes in health for two reasons. First, there was a relatively large fall in employment level among the older population in the UK, compared with their younger counterparts (Powell et al. 2022). Second, older adults have been shown to be more resilient to anxiety, depression, and other stress-related disorders seen among younger populations during the early stages of the pandemic (Vahia et al. 2020). Therefore, the direct effect of financial insecurity may be larger in magnitude than their indirect effect, leading to hypothesis 3:

- Hypothesis 3. People with financial insecurity are more likely to retire later, or less likely to retire earlier.

## Data and methods

The English Longitudinal Study of Ageing (ELSA) is an ongoing panel study representing men and women aged 50 + who reside in private households in England. The study began in 2002 (Wave 1), with responses from 12,099 individuals comprising core members (those representative of the English population aged 50+) and their partners. Every two years, sample participants are interviewed on their health, social, psychological, cognitive, and economic circumstances; in addition, every four years, nurse visits are conducted for the collection of biological samples and anthropometric measurements. The most recent sweep of the study was Wave 9, with data collection spanning June 2018 and July 2019. The sample was also refreshed at Waves 3, 4, 6, 7 and 9, to ensure the sample remains nationally representative (Steptoe et al. 2013).

### Sample

The ELSA COVID-19 Substudy, administered between June 3 and July 26, 2020 (Wave 1) and again between November 4 and December 20, 2020 (Wave 2), was also used. The Wave 1 (Wave 2) survey was issued to 9525 (9150) eligible members, with 7040 (6794) interviews completed, achieving a 74% response rate. In both waves, the survey was administered online (83%) or by telephone interview for those who were not able to respond online (17%). As our analysis relied on some key information collected prior to the pandemic, our sample comprised only core members who were also observed in ELSA Wave 9 (*n* = 5583 in COVID-19 Wave 1, *n* = 5148 in COVID-19 Wave 2) (Addario et al. 2020). In addition, participants who reported themselves to be retired, permanently sick or disabled, or looking after their home or family were excluded from our sample, which resulted in a considerable drop in sample size. The final weighted working sample consisted of 1354 interviews in Wave 1 and 1201 interviews in Wave 2 with non-missing information on the key variables (detailed below), with the weights adjusting for non-response in the corresponding COVID-19 Wave, contingent on response in ELSA Wave 9.

### Outcome variable

In both waves of COVID-19 Substudy, participants were asked, “Has the age at which you expect to retire from paid work changed as a result of the coronavirus outbreak?”, with response options “Yes, I now plan to retire earlier”, “Yes, I now plan to retire later”, and “No”. Three groups of changes in retirement plans were constructed accordingly.

### Key exposures

The focus of this study was on two main areas of exposure during the COVID-19 pandemic: financial insecurity and health. For financial insecurity, participants were asked to rate on a five-point scale, how worried they were, if at all, about their future financial situation. This variable was dichotomised to indicate financial insecurity (i.e., somewhat, very, or extremely worried, vs. not at all or not very worried). They were also asked to rate on a five-point scale, how their current financial situation compared to before the coronavirus outbreak. This variable was collapsed into three categories, namely (a little or much) better off, about the same, and (a little or much) worse off.

As for health, participants were asked to rate on a five-point scale, how they would say their health was in the past month. This variable was dichotomised to indicate poor self-rated general health (i.e., fair or poor, vs. excellent, very good, or good). The measurement of mental health was based on eight items from the Center for Epidemiologic Studies Depression (CES-D) scale, which measured participants’ depressive symptoms in the week prior to interview (Beekman et al. 1997). Participants were categorised as experiencing depressive symptomology if they responded positively to four or more symptoms (Zaninotto et al. 2022).

### Covariates

The statistical analysis adjusted for pertinent pre-pandemic covariates taken from information collected in Wave 9. This included gender, age, ethnicity (white vs. otherwise), partnership status (married or cohabiting, vs. otherwise), whether they have dependent children, and whether they live in an urban or rural area. Past health information was also considered, namely whether they reported a limiting long-term illness and depressive symptomology, the latter captured with the same measure used in COVID-19 Waves 1 and 2.

For economic conditions, only pre-pandemic adjustments were available, including participants’ education (degree vs. otherwise), social class (managerial, administrative, and professional occupations; vs. intermediate occupations, small employers, and own account workers; vs. lower supervisory, technical, semi-routine, and routine occupations), neighbourhood deprivation levels (captured using Index of Multiple Deprivation quintiles), experience of financial difficulties (not managing very well financially, or have some or severe financial difficulties; vs. getting by alright financially, or managing quite or very well financially), home ownership status, and wealth levels.

From the COVID-19 Waves, covariates included whether they had private pensions from which they had not yet started receiving or drawing an income, and whether they were working at the time of interview (i.e., currently working; vs. on paid or unpaid leave from employment including furlough, or self-employed but not currently working), as well as whether participants had any experience of COVID-19, including having tested positive for COVID-19 (themselves, a household member, or someone close to them outside their household), stayed in hospital for treatment due to COVID-19 (themselves or a household member) or died from COVID-19 (a household member or someone close to them outside their household).

### Analytical strategy

Multinomial logistic specifications of changes in retirement plans (using “no change” as reference) were estimated separately for COVID-19 Waves 1 and 2. In our longitudinal analysis, a random-effect model was estimated to account for unobserved individual heterogeneity (weighted to account for non-response in the COVID-19 waves, contingent on response in ELSA Wave 9), and a likelihood-ratio test informed the use of an independent covariance structure (cf. unstructured covariance). Exponentiated coefficient estimates were interpreted as relative risk ratios (RRRs). To rule out potential selection bias associated with sample exclusion, inverse probability weighting (IPW) was applied to cross-sectional multinomial logistic regressions, in a selectivity analysis. All analyses were conducted using Stata 17.0.

## Results

### Descriptive statistics

In June/July 2020, among older adults in the labour force (i.e., employed, on paid or unpaid leave from employment, or self-employed and working or not working), around 4.9% were planning to retire earlier due to the pandemic, 8.8% were planning to retire later, with the remainder (86.3%) reporting no change in their expected age at retirement. By November/December 2020, 7.3% were reportedly planning to retire earlier due to the pandemic, and 11.8% were planning to retire later. Characteristics of participants by these changes in retirement plans in June/July and November/December 2020 are shown in Supplementary Tables A1 and A2, respectively.

**Table 1:** Cross-sectional multinomial logistic regressions

Ref: no change	Covid Wave 1 (Jun/Jul 2020)				Covid Wave 2 (Nov/Dec 2020)			
	Retiring earlier		Retiring later		Retiring earlier		Retiring later	
*Main exposure*								
Poor self-rated health	1.043	(0.491)	1.953*	(0.606)	0.687	(0.302)	0.513	(0.183)
Depressive symptomatology	0.860	(0.363)	1.293	(0.406)	1.386	(0.583)	1.862*	(0.472)
Worried about future financial situation	0.794	(0.273)	2.154**	(0.598)	0.556	(0.200)	2.003**	(0.521)
*Controls from 2018/19*								
Male	0.811	(0.244)	1.621	(0.404)	0.928	(0.262)	1.452	(0.340)
Age	1.034	(0.028)	1.021	(0.024)	0.987	(0.025)	1.017	(0.026)
Non-white	1.790	(0.955)	1.812	(0.723)	3.982**	(2.064)	1.368	(0.583)
Partnered	2.538*	(0.973)	0.877	(0.245)	1.197	(0.381)	1.041	(0.301)
Have children in benefit unit	0.840	(0.375)	1.431	(0.516)	0.791	(0.341)	1.320	(0.408)
Live in rural area	0.634	(0.230)	0.898	(0.257)	0.451*	(0.164)	1.598	(0.407)
Limiting, long-term illness	0.866	(0.368)	0.604	(0.243)	0.989	(0.410)	0.555	(0.208)
Depressive symptomology	1.124	(0.664)	1.140	(0.497)	0.284	(0.248)	1.960	(0.793)
Degree [NVQ4-5]	0.960	(0.299)	1.578	(0.431)	0.640	(0.208)	1.297	(0.354)
Social class								
Managerial, administrative, professional								
Intermediate	0.992	(0.507)	0.904	(0.367)	0.604	(0.289)	0.672	(0.260)
Routine/manual	0.745	(0.332)	1.079	(0.432)	0.965	(0.403)	1.196	(0.420)
Other/incomplete info	1.256	(0.459)	1.244	(0.453)	0.673	(0.256)	1.114	(0.351)
Index of Multiple Deprivation								
Quintile 1 (least deprived)								
Quintile 2	0.824	(0.295)	1.014	(0.363)	1.132	(0.420)	1.088	(0.332)
Quintile 3	0.296**	(0.135)	1.010	(0.408)	0.699	(0.288)	0.805	(0.272)
Quintile 4	0.524	(0.222)	1.887	(0.736)	0.535	(0.225)	1.081	(0.404)
Quintile 5 (most deprived)	0.424	(0.269)	0.417	(0.219)	0.743	(0.404)	0.748	(0.401)
Financial difficulties	0.859	(0.575)	0.914	(0.358)	0.294	(0.253)	0.511	(0.195)
Own home	2.049*	(0.644)	1.232	(0.349)	1.849*	(0.579)	0.776	(0.204)
Log wealth	0.984	(0.043)	1.011	(0.025)	1.036	(0.071)	0.999	(0.026)
*Controls from 2020*								
Have private pension	2.263*	(0.718)	1.560	(0.428)	1.366	(0.385)	1.284	(0.315)
Currently working	1.672	(0.835)	0.828	(0.293)	0.280**	(0.120)	1.270	(0.531)
Financial condition due to COVID								
Better off	1.271	(0.476)	1.646	(0.643)	1.504	(0.528)	2.088*	(0.652)
Same (ref.)								
Worse off	2.072*	(0.749)	3.163**	(0.927)	1.731	(0.616)	3.399**	(0.940)
Covid exposure	1.290	(0.423)	1.014	(0.314)	1.143	(0.304)	0.831	(0.205)
*N*	1354				1201			

Figures are relative-risk ratios. *p < 0.05, **p < 0.01

**Table 2:** Multinomial logistic regressions with interactions

Ref: no change	Covid Wave 1 (Jun/Jul 2020)								Covid Wave 2 (Nov/Dec 2020)							
	Retiring earlier				Retiring later				Retiring earlier				Retiring later			
*(A) Self-rated health*																
Financial insecurity	0.794	(0.273)	0.744	(0.271)	2.154**	(0.598)	1.633	(0.527)	0.556	(0.200)	0.547	(0.213)	2.003**	(0.521)	2.030**	(0.529)
Poor health	1.043	(0.491)	0.846	(0.478)	1.953*	(0.606)	0.433	(0.273)	0.687	(0.302)	0.662	(0.334)	0.513	(0.183)	0.567	(0.363)
Interaction^a^			1.566	(1.388)			6.898**	(4.701)			1.103	(0.947)			0.876	(0.633)
*(B) Depressive symp.*																
Financial insecurity	0.794	(0.273)	0.928	(0.344)	2.154**	(0.598)	1.846*	(0.566)	0.556	(0.200)	0.604	(0.256)	2.003**	(0.521)	1.760	(0.511)
Depressive symp.	0.860	(0.363)	1.231	(0.635)	1.293	(0.406)	0.603	(0.452)	1.386	(0.583)	1.521	(0.692)	1.862*	(0.472)	1.349	(0.643)
Interaction^b^			0.404	(0.348)			2.721	(2.478)			0.781	(0.558)			1.608	(0.916)

All panels represent different regressions. Each regression includes the same set of controls and modifiers as in Table 1. *p < 0.05, **p < 0.01

^a^Interaction term between financial insecurity and poor health.

^b^Interaction term between financial insecurity and depressive symptomatology

**Fig. 1:**
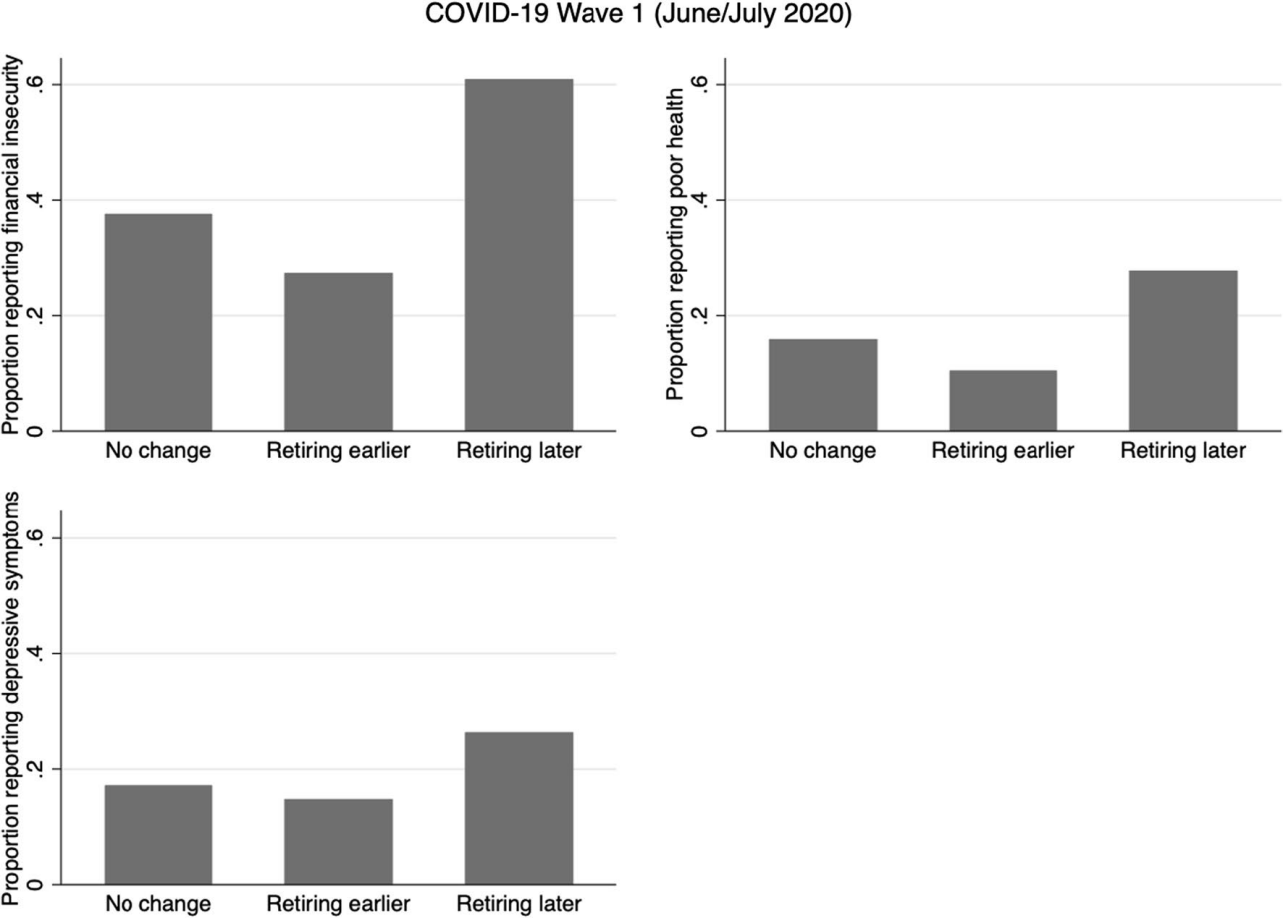
Prevalence of financial insecurity, poor health, and depressive symptomology, by changes in retirement plans, in COVID-19 Wave 1

**Fig. 2:**
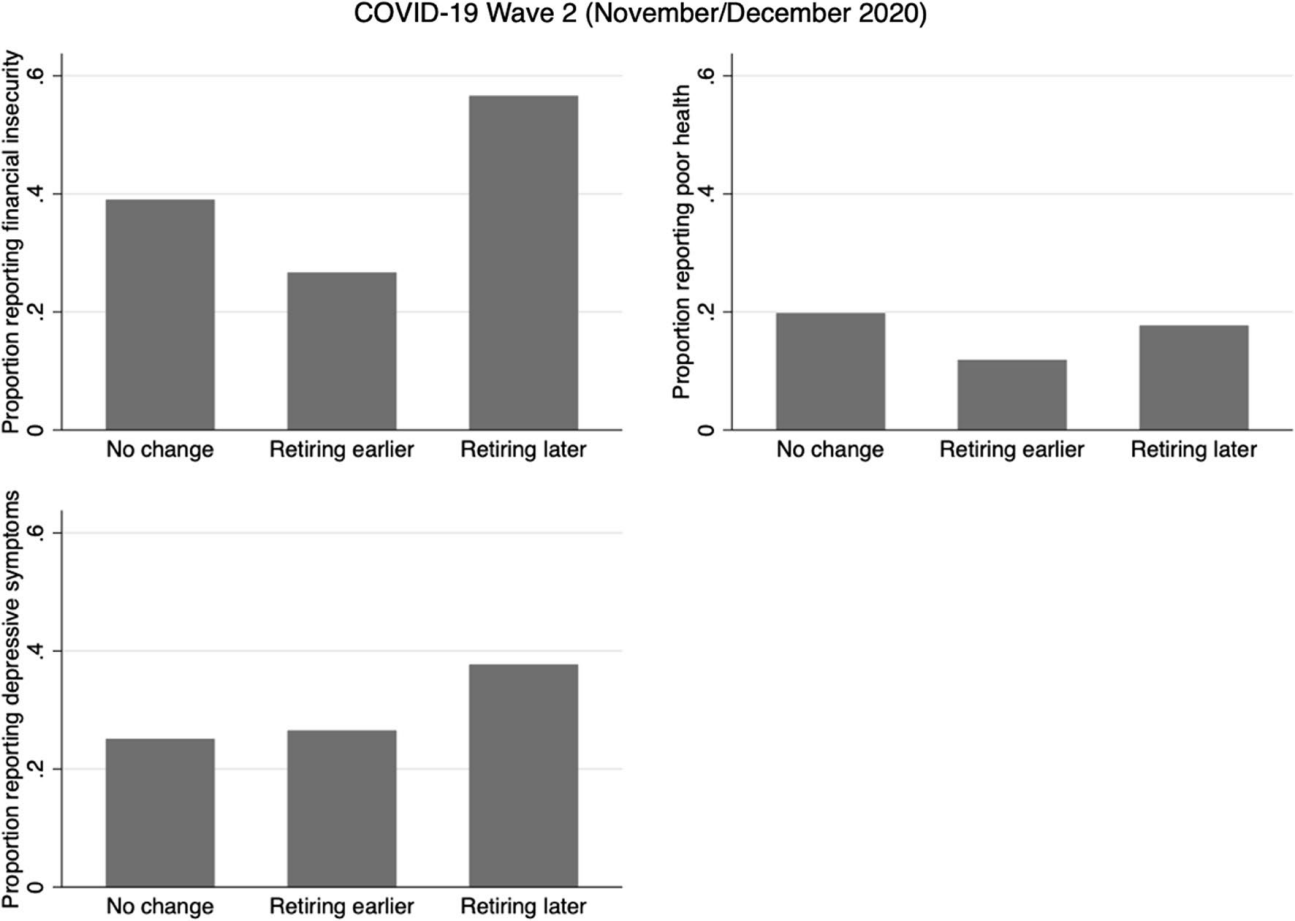
Prevalence of financial insecurity, poor health, and depressive symptomology, by changes in retirement plans, in COVID-19 Wave 2

In general, compared with older adults reporting no change in retirement plans, those planning to retire earlier were less likely to be worried about their future financial situation, whereas those planning to retire later were more likely to be worried about their future financial situation. Those who in June/July 2020 reported a plan to retire later were also more likely to being financially worse off as a consequence of the pandemic. By November/December 2020, those who reported planning to retire earlier were also more likely to be in poor health, whereas those planning to retire later were more likely to have depressive symptomology (Figs. 1 and 2).

Looking at demographic characteristics observed in 2018/19 (ELSA Wave 9), compared with older adults who reported no change in retirement plans due to the pandemic, those reporting in June/July 2020 a plan to retire earlier were more likely to be partnered. In November/December 2020, those reporting a plan to retire earlier were more likely to live in an urban area and to experience depressive symptomology. Little difference in demographic characteristics, such as sex, age, education, and household composition, was observed between those planning to retire later and those reporting no change in plans, across both COVID-19 periods.

As for pre-pandemic economic conditions, older adults reporting in June/July 2020 a plan to retire earlier were wealthier, and more likely to live in a better (i.e., less deprived) neighbourhood, own their own home and have a private pension, compared with those reporting no change in plans. In contrast, older adults planning to retire later were more likely to live in a more deprived neighbourhood. Less of a difference was observed in pre-pandemic economic conditions by retirement plans reported in November/December 2020, except that those planning to retire earlier were, notably, economically better off.

### Cross-sectional analysis

Results from the separate multinomial logistic regressions for the COVID-19 waves are presented in Table 1—figures are relative risk ratios (RRRs), or risks in relation to the reference of no change in retirement plans.

#### Health

After adjusting for covariates, older adults reporting poor health were relatively more likely to plan to retire later over not changing their plans, compared to those reporting better health, but this was only evident when interviewed in June/July 2020 (RRR: 1.953). Hypothesis 1 was rejected. By November/December, older adults reporting depressive symptomology were more likely to plan to retire later over not changing their plans (RRR: 1.862). Hypothesis 2 was rejected.

#### Financial insecurity

Consistent with the descriptive statistics, regression estimates showed that financial insecurity was significantly predictive of plans to retire later due to the pandemic, even after adjusting for pre-pandemic financial difficulties: the RRR in June/July 2020 was 2.154. Therefore, on average, the relative risk of planning to retire later over not changing retirement plans among those who were worried about their future financial situation, was around twice as high as this relative risk among those who were not worried. This influence was more likely to be directly related to the pandemic rather than general effects. Moreover, this estimated relationship remained significant in November/December 2020, where the RRR was 2.003. Hypothesis 3 cannot be rejected.

#### Covariates

Notably, in June/July 2020, older adults who were married or cohabiting, lived in a less deprived neighbourhood, owned their home and had private pension, were relatively more likely to report planning to retire earlier over not changing their retirement plans, compared with their respective counterparts. In contrast, by November/December 2020, predictive characteristics were identifying as non-white, living in an urban area, owning their own home and not working at the time of interview (i.e., paid/unpaid leave from employment, or self-employed but not currently working). No significant association was found between social class and changes in retirement plans, possibly due to the inclusion of home ownership and individual wealth which captured lifetime accumulated wealth. Across both COVID-19 Waves, older adults with depressive symptomology in 2018/19 were more likely to report planning to retire later over not changing plans, than those who did not experience these symptoms previously. Experience of COVID-19 was not predictive of changes in retirement plans at either COVID-19 Wave.

Results from a selectivity analysis applying inverse probability weighting to these cross-sectional multinomial logistic regressions were consistent, with similar magnitudes (see Supplementary Table A3).

**Table 3:** Panel multinomial logistic regressions

Ref: no change	Random effects with zero covariance			
	Retiring earlier		Retiring later	
*Main exposure*				
Poor self-rated health	0.634	(0.277)	0.972	(0.421)
Depressive symptomology	1.110	(0.393)	1.657	(0.581)
Worried about future financial situation	0.622	(0.217)	3.315**	(1.127)
*Controls from 2018/19*				
Male	0.749	(0.232)	1.850	(0.594)
Age	1.015	(0.029)	1.019	(0.035)
Non-white	4.373*	(2.681)	1.670	(1.012)
Partnered	1.984	(0.753)	0.933	(0.357)
Have children in benefit unit	0.781	(0.377)	1.365	(0.614)
Live in rural area	0.387*	(0.154)	1.530	(0.568)
Limiting, long-term illness	1.150	(0.476)	0.441	(0.237)
Depressive symptomology	0.326	(0.226)	2.342	(1.423)
Degree [NVQ4-5]	0.618	(0.217)	1.645	(0.628)
Social class				
Managerial, administrative, profsessional				
Intermediate	0.578	(0.322)	0.906	(0.505)
Routine/manual	0.806	(0.370)	1.434	(0.666)
Other/incomplete info	0.757	(0.300)	1.561	(0.668)
Index of Multiple Deprivation				
Quintile 1 (least deprived)				
Quintile 2	0.939	(0.385)	1.205	(0.509)
Quintile 3	0.374*	(0.174)	0.907	(0.426)
Quintile 4	0.390*	(0.185)	1.519	(0.767)
Quintile 5 (most deprived)	0.443	(0.250)	0.417	(0.287)
Financial difficulties	0.447	(0.297)	0.497	(0.267)
Own home	2.316*	(0.793)	0.822	(0.287)
Log wealth	1.000	(0.000)	1.000	(0.000)
*Controls from 2020*				
Have private pension	2.148*	(0.719)	1.744	(0.583)
Currently working	0.427	(0.200)	1.258	(0.641)
*Modifiers*				
Financial condition due to COVID				
Better off	1.257	(0.482)	1.687	(0.671)
Same (ref.)				
Worse off	1.764	(0.642)	4.011**	(1.206)
Covid exposure	1.108	(0.348)	0.980	(0.336)
var(u1)	115.753** (207.922)			
var(u2)	972.068** (2588.136)			
Wave dummy included	Yes			
*N*	1319			

Figures are relative-risk ratios. *p < 0.05, **p < 0.01

#### Interaction effects of health and financial insecurity

Given the significant roles of the exposure variables, particularly for the relative risk of planning to retire later over not changing retirement plans (hereafter simply referred to as ‘relative risk’ in the rest of this subsection), Table 2 provides further estimations from interacting financial insecurity with each of the other exposures of interest, while still controlling for all other covariates as in Table 1.

After including the (nonsignificant, positive) interaction term between financial insecurity and depressive symptomology, both their main effects were no longer predictive of this relative risk, across both time points. This was also partly the case for self-rated health: in June/July 2020, both the main effects of financial insecurity and health were no longer significant after the inclusion of their interaction term which was, in turn, strongly predictive of a higher relative risk. The RRR estimate of 6.898 suggests that the relative risk was higher by nearly seven times among those experiencing both financial insecurity and poor self-rated health, than among those experiencing neither. By November/December, this interaction term was no longer significant, and the main effect of financial insecurity remained significant. Results were consistent when inverse probability weighting was applied, reported in Supplementary Table A4.

### Longitudinal analysis

Table 3 provides results from estimating the multinomial logit regression in a panel setup for the two COVID-19 waves, thereby accounting for unobserved heterogeneity at the individual level. Results from a likelihood-ratio test validated the assumption of zero covariance.

Consistent with the cross-sectional estimates at both time points in Table 1, neither health nor financial insecurity were significantly predictive of the relative risk of planning to retire earlier over not changing retirement plans. Significant relationships were observed between financial insecurity and the relative risk of planning to retire later over not changing retirement plans (RRR: 3.315). However, self-rated health was no longer predictive of this relative risk.

Consistent with Table 1, the demographic and economic covariates captured from 2018/19 (ELSA Wave 9) played a larger role for the relative risk of planning to retire earlier over not changing retirement plans, than for the relative risk of planning to retire later. Identifying as non-white, living in an urban area, having no past depressive symptomology, living in a less deprived area, having their own home, having private pension, and not currently working, were predictive of a higher relative risk of planning to retire earlier over not changing retirement plans. On the other hand, only having past depressive symptomology predicted a higher relative risk of planning to retire later.

In the interaction analysis between financial insecurity with each of the other exposures of interest (Supplementary Table A5), very little impact of the interaction terms was observed; instead, persistent and strong main effects of financial insecurity due to COVID-19 were found on the relative risk of planning to retire later over not changing retirement plans.

## Discussion

The aim of this article was to examine how health factors and financial insecurity affected older adults’ retirement plans during the COVID-19 pandemic in the UK. It was found that poor self-rated health was related to a higher risk of postponing retirement (relative to not changing retirement plans) when interviewed in June/July 2020, but a lower risk of postponing retirement, when interviewed later in November/December 2020. A positive association between depressive symptomology and risk of postponing retirement was only pronounced in November/December 2020. Financial insecurity was associated with a higher risk of postponing retirement at both timepoints. However, plans of retiring earlier were not affected by these health factors or financial insecurity. These findings can be broadly generalised among English adults aged 50 and older in the context of the COVID-19 pandemic.

In the first months of the pandemic, people with poor self-rated health had a stronger intention of postponing retirement than people reporting good or fair health. Despite having poor health, people would not leave their jobs during the pandemic, which was different from previous findings (e.g., Von Bonsdorff et al. 2010; Gørtz 2012; Scharn et al. 2018). It might be because people with poor health tend to be more risk averse (Decker and Schmitz 2016; Courbage et al. 2018). Faced with some negative socio-economic consequences of the pandemic, such as high unemployment rate and fewer vacancies (Mayhew & Anand 2020; Arthur 2021), they may have been more likely to postpone their retirement, to stay in the labour market to make a living. In the present study, older adults in poor health were more risk averse regarding financial circumstances than those in good health (score of 3.1 vs. 3.7, on a scale from 0 ‘avoid taking risks’ to 10 ‘fully prepared to take risks’, when observed in 2016/17). This explanation is also supported by the results of the interaction analysis showing that among older adults who were financially secure, those reporting poor health did not tend to change their retirement plans.

Another possible explanation is that self-rated healthier adults were trying to devote more time to leisure activities and to be with their families, rather than to work, after experiencing the outbreak of the pandemic and the first national lockdown in the UK, with the expectation that the pandemic was a short-lived event. In contrast, by November/December 2020, people may have realised that the pandemic would last longer than expected, and started to adapt to a ‘new normal’ (Corpuz 2021), so changes in retirement plans of healthier adults were not pronounced. However, poor self-rated health was associated with a lower possibility of delaying retirement, consistent with the literature that poor health pushes people out of their jobs (Mein et al. 2000; Homaie Rad et al. 2017).

The impact of mental health on retirement plans was only pronounced in November/December 2020, in line with the finding of greater depression among ELSA participants in November/December than in June/July 2020 (Zaninotto et al. 2022). Elevated depressive symptomatology almost doubled the risk of postponing retirement (relative to not changing retirement plans). This is plausible, since people with fewer depressive symptoms are more likely to be optimistic (Conversano et al. 2010; Galatzer-Levy and Bonanno 2014; Hobbs et al. 2022). They are also more confident about the future (Carver et al. 2010), including potentially expecting an eventual upturn of their financial situation or having a stronger belief in their financial capabilities. In the present study, older adults with fewer depressive symptoms were more likely than those with depression symptomatology to ‘feel hopeful’ (71% vs. 49%, measured in 2010/11), to regard ageing as a positive experience (68% vs. 49%, measured in 2016/17), to report that they would change almost nothing if they could live their life again (61% vs. 36%, measured in 2018/19), to feel that life is full of opportunities (49% vs. 26%, measured in 2018/19), and to feel that the future looks good for them (54% vs. 24%, measured in 2018/19).

Compared with older adults who were not worried about their future financial situation, those reporting financial insecurity were more likely to plan to retire later at both timepoints, in line with the literature (Mein et al. 2000; Oksanen and Virtanen 2012; Van Droogenboeck and Spruyt 2014). Consistent results were found in the longitudinal multinomial logistic analysis. Put together, these findings suggest that financial insecurity was a stable and persistent predictor of changes in retirement plans during the pandemic. Our interaction analysis also showed an additional influence of financial insecurity on the risk of postponing retirement among adults with poor self-rated health, compared with those with good health. It might be because the receipt of disability benefits increased the likelihood of earlier retirement plans (Börsch-Supan et al. 2009; Autor et al. 2016). In the present study, among older adults who reported poor health, 11.6% of those who were financially insecure were receiving disability benefits, which is a smaller proportion than those reporting to be financially secure (13.9%). In contrast, among those reporting good health, 4.9% of those experiencing financial insecurity were receiving disability benefits, which is more than those reporting to be financially secure (3%).

Our findings should nonetheless be interpreted in the light of several limitations. First, due to the restricted observation period, the estimates of this study reveal changes in retirement plans among English older adults after experiencing the first national lockdown over March-July 2020 and the second national lockdown over November–December 2020 in England, rather than after experiencing the entire COVID-19 pandemic over 2020–2022. Second, even though self-rated health is typically used in the literature as a proxy for health, this subjective health measure may be endogenous and biased (Oksanen and Virtanen 2012). Third, older adults’ actual retirement behaviour was not observed, and the findings were limited to self-reported changes in retirement plans. Finally, further research is needed to investigate differences in the impact of health factors and financial insecurity on older adults’ changes in retirement plans, such as occupational differences.

In conclusion, this study highlights the critical role of financial insecurity on older adults’ retirement decisions, even when facing a crisis that impacts multiple dimensions of their lives. Poor health and depressive symptomology played different roles in the two periods observed, which could be an artefact of the fluctuating nature of the crisis—one period was towards the end of the first national lockdown, whereas the other included not only the beginning of the second national lockdown but also some of the earliest days of the vaccine rollout—suggesting a more contextual role of health in retirement planning. Importantly, these findings may aid in informing decisions on budgets, risk management, and caring for the health of older workers, among government, private pension providers, and firms with an older workforce.

## Supplementary Information

Below is the link to the electronic supplementary material.

Supplementary file 1 (DOCX 42 kb)
