# Supplementary material for: Changes in retirement plans in the English older population during the COVID-19 pandemic: The roles of health factors and financial insecurity
Source: Eur J Ageing. 2023 Jun 13;20(1):22. doi: 10.1007/s10433-023-00770-1 (PMC10262925; doi:10.1007/s10433-023-00770-1)
Supplement: Supplementary file 1 — (DOCX 42 kb) [file 10433_2023_770_MOESM1_ESM.docx]

**Table A1.** Descriptive statistics: retirement plan at COVID-19 Wave 1 (Jun/Jul 2020)

| Dependent variable | No change | | Retiring earlier | | Retiring later | | Total sample | |
| --- | --- | --- | --- | --- | --- | --- | --- | --- |
| **Main exposure** |  |  |  |  |  |  |  |  |
| Worried about future financial situation | 0.371 | (0.017) | 0.275 | (0.061) | 0.614 | (0.058) | 0.387 | (0.016) |
|  |  |  |  |  |  |  |  |  |
| **Controls from 2018/19** |  |  |  |  |  |  |  |  |
| Male | 0.513 | (0.018) | 0.513 | (0.068) | 0.620 | (0.056) | 0.522 | (0.016) |
| Age | 58.512 | (0.152) | 59.168 | (0.652) | 58.202 | (0.406) | 58.516 | (0.140) |
| Non-white | 0.103 | (0.013) | 0.114 | (0.045) | 0.155 | (0.054) | 0.108 | (0.013) |
| Partnered | 0.720 | (0.016) | 0.880 | (0.040) | 0.703 | (0.054) | 0.726 | (0.015) |
| Have children in BU | 0.159 | (0.012) | 0.136 | (0.048) | 0.195 | (0.047) | 0.161 | (0.012) |
| Live in rural area | 0.228 | (0.014) | 0.169 | (0.048) | 0.231 | (0.047) | 0.226 | (0.013) |
| Limiting, long-term illness | 0.168 | (0.013) | 0.148 | (0.045) | 0.154 | (0.042) | 0.166 | (0.012) |
| Depressive symptomology | 0.086 | (0.010) | 0.066 | (0.031) | 0.112 | (0.040) | 0.088 | (0.010) |
| Has degree [NVQ4-5] | 0.240 | (0.014) | 0.283 | (0.059) | 0.338 | (0.056) | 0.251 | (0.013) |
| Social class |  |  |  |  |  |  |  |  |
| Manager, admin, prof. | 0.209 | (0.013) | 0.225 | (0.053) | 0.203 | (0.047) | 0.209 | (0.012) |
| Intermediate | 0.150 | (0.012) | 0.144 | (0.048) | 0.126 | (0.040) | 0.148 | (0.011) |
| Routine/manual | 0.205 | (0.014) | 0.149 | (0.049) | 0.192 | (0.048) | 0.201 | (0.013) |
| Other/incomplete info | 0.435 | (0.018) | 0.482 | (0.068) | 0.479 | (0.060) | 0.442 | (0.016) |
| Index of Multiple Dep. |  |  |  |  |  |  |  |  |
| Quintile 1 (least dep.) | 0.217 | (0.014) | 0.368 | (0.066) | 0.200 | (0.045) | 0.223 | (0.013) |
| Quintile 2 | 0.225 | (0.014) | 0.304 | (0.062) | 0.207 | (0.043) | 0.227 | (0.013) |
| Quintile 3 | 0.222 | (0.014) | 0.104 | (0.039) | 0.196 | (0.048) | 0.214 | (0.013) |
| Quintile 4 | 0.188 | (0.014) | 0.142 | (0.049) | 0.332 | (0.059) | 0.199 | (0.014) |
| Quintile 5 (most dep.) | 0.148 | (0.014) | 0.082 | (0.042) | 0.065 | (0.034) | 0.138 | (0.012) |
| Financial difficulties | 0.129 | (0.013) | 0.070 | (0.033) | 0.167 | (0.050) | 0.130 | (0.012) |
| Own home | 0.410 | (0.017) | 0.606 | (0.067) | 0.410 | (0.056) | 0.419 | (0.016) |
| Wealth | 370143 | (15693) | 471161 | (59750) | 399315 | (56954) | 377653 | (14748) |
|  |  |  |  |  |  |  |  |  |
| **Controls from 2020** |  |  |  |  |  |  |  |  |
| Have private pension | 0.495 | (0.018) | 0.690 | (0.060) | 0.607 | (0.060) | 0.514 | (0.016) |
| Currently working | 0.879 | (0.012) | 0.935 | (0.029) | 0.814 | (0.049) | 0.876 | (0.011) |
|  |  |  |  |  |  |  |  |  |
| **Modifiers** |  |  |  |  |  |  |  |  |
| Financial condition due to COVID |  |  |  |  |  |  |  |  |
| Better off | 0.200 | (0.014) | 0.233 | (0.056) | 0.189 | (0.051) | 0.201 | (0.013) |
| Same (ref.) | 0.531 | (0.017) | 0.420 | (0.068) | 0.285 | (0.053) | 0.504 | (0.016) |
| Worse off | 0.269 | (0.016) | 0.347 | (0.065) | 0.526 | (0.060) | 0.296 | (0.015) |
| Depressive symptomatology | 0.176 | (0.014) | 0.145 | (0.046) | 0.251 | (0.056) | 0.181 | (0.013) |
| Poor self-rated health | 0.161 | (0.014) | 0.112 | (0.040) | 0.273 | (0.055) | 0.168 | (0.013) |
| Covid exposure | 0.225 | (0.015) | 0.274 | (0.060) | 0.245 | (0.055) | 0.230 | (0.014) |
| *N* | 1175 |  | 74 |  | 105 |  | 1354 |  |
| *Weighted proportion* | 0.863 |  | 0.049 |  | 0.088 |  |  |  |

**Table A2.** Descriptive statistics: retirement plan at COVID-19 Wave 2 (Nov/Dec 2020)

| Dependent variable | No change | | Retiring earlier | | Retiring later | | Total sample | |
| --- | --- | --- | --- | --- | --- | --- | --- | --- |
| **Main exposure** |  |  |  |  |  |  |  |  |
| Worried about future financial situation | 0.389 | (0.019) | 0.250 | (0.058) | 0.584 | (0.051) | 0.402 | (0.017) |
|  |  |  |  |  |  |  |  |  |
| **Controls from 2018/19** |  |  |  |  |  |  |  |  |
| Male | 0.506 | (0.019) | 0.530 | (0.065) | 0.603 | (0.049) | 0.519 | (0.017) |
| Age | 58.441 | (0.162) | 58.825 | (0.531) | 57.861 | (0.390) | 58.400 | (0.144) |
| Non-white | 0.095 | (0.015) | 0.166 | (0.063) | 0.134 | (0.044) | 0.105 | (0.014) |
| Partnered | 0.717 | (0.018) | 0.809 | (0.050) | 0.754 | (0.045) | 0.728 | (0.016) |
| Have children in BU | 0.144 | (0.013) | 0.127 | (0.041) | 0.227 | (0.041) | 0.153 | (0.012) |
| Live in rural area | 0.223 | (0.015) | 0.117 | (0.037) | 0.300 | (0.046) | 0.225 | (0.013) |
| Limiting, long-term illness | 0.176 | (0.015) | 0.144 | (0.044) | 0.132 | (0.030) | 0.169 | (0.013) |
| Depressive symptomology | 0.093 | (0.013) | 0.027 | (0.017) | 0.151 | (0.041) | 0.095 | (0.012) |
| Has degree [NVQ4-5] | 0.251 | (0.016) | 0.230 | (0.049) | 0.320 | (0.048) | 0.257 | (0.014) |
| Social class |  |  |  |  |  |  |  |  |
| Manager, admin, prof. | 0.207 | (0.014) | 0.264 | (0.053) | 0.231 | (0.040) | 0.214 | (0.013) |
| Intermediate | 0.159 | (0.013) | 0.129 | (0.042) | 0.099 | (0.033) | 0.150 | (0.012) |
| Routine/manual | 0.199 | (0.016) | 0.198 | (0.047) | 0.194 | (0.042) | 0.198 | (0.014) |
| Other/incomplete info | 0.435 | (0.019) | 0.410 | (0.068) | 0.475 | (0.052) | 0.438 | (0.017) |
| Index of Multiple Dep. |  |  |  |  |  |  |  |  |
| Quintile 1 (least dep.) | 0.215 | (0.015) | 0.287 | (0.058) | 0.232 | (0.041) | 0.222 | (0.014) |
| Quintile 2 | 0.215 | (0.015) | 0.283 | (0.058) | 0.256 | (0.042) | 0.225 | (0.014) |
| Quintile 3 | 0.232 | (0.016) | 0.187 | (0.057) | 0.205 | (0.040) | 0.226 | (0.015) |
| Quintile 4 | 0.193 | (0.016) | 0.130 | (0.043) | 0.228 | (0.049) | 0.193 | (0.014) |
| Quintile 5 (most dep.) | 0.144 | (0.015) | 0.113 | (0.040) | 0.078 | (0.034) | 0.134 | (0.013) |
| Financial difficulties | 0.150 | (0.016) | 0.043 | (0.026) | 0.133 | (0.044) | 0.140 | (0.014) |
| Own home | 0.404 | (0.018) | 0.580 | (0.065) | 0.333 | (0.046) | 0.408 | (0.017) |
| Wealth | 368290 | (17268) | 515356 | (82557) | 397450 | (40080) | 382422 | (16048) |
|  |  |  |  |  |  |  |  |  |
| **Controls from 2020** |  |  |  |  |  |  |  |  |
| Have private pension | 0.503 | (0.019) | 0.590 | (0.066) | 0.573 | (0.053) | 0.518 | (0.017) |
| Currently working | 0.904 | (0.012) | 0.826 | (0.045) | 0.893 | (0.037) | 0.897 | (0.011) |
|  |  |  |  |  |  |  |  |  |
| **Modifiers** |  |  |  |  |  |  |  |  |
| Financial condition due to COVID |  |  |  |  |  |  |  |  |
| Better off | 0.195 | (0.015) | 0.251 | (0.064) | 0.222 | (0.044) | 0.202 | (0.014) |
| Same (ref.) | 0.563 | (0.019) | 0.444 | (0.064) | 0.319 | (0.047) | 0.525 | (0.017) |
| Worse off | 0.242 | (0.017) | 0.305 | (0.060) | 0.458 | (0.052) | 0.272 | (0.016) |
| Depressive symptomatology | 0.260 | (0.018) | 0.227 | (0.053) | 0.404 | (0.051) | 0.275 | (0.016) |
| Poor self-rated health | 0.197 | (0.017) | 0.117 | (0.037) | 0.158 | (0.035) | 0.187 | (0.014) |
| Covid exposure | 0.334 | (0.018) | 0.372 | (0.062) | 0.286 | (0.045) | 0.331 | (0.016) |
| *N* | 981 |  | 84 |  | 136 |  | 1201 |  |
| *Weighted proportion* | 0.809 |  | 0.073 |  | 0.118 |  |  |  |

**Table A3.** Cross-sectional multinomial logistic regressions using inverse probability weighting

|  | Covid Wave 1 (Jun/Jul 2020) | | | | Covid Wave 2 (Nov/Dec 2020) | | | |
| --- | --- | --- | --- | --- | --- | --- | --- | --- |
| Ref: no change | Retiring earlier | | Retiring later | | Retiring earlier | | Retiring later | |
| **Main exposure** |  |  |  |  |  |  |  |  |
| Poor self-rated health | 1.044 | (0.505) | 1.898* | (0.597) | 0.626 | (0.278) | 0.487* | (0.175) |
| Depressive symptomatology | 0.865 | (0.375) | 1.348 | (0.427) | 1.438 | (0.610) | 1.866* | (0.481) |
| Worried about future financial situation | 0.808 | (0.272) | 2.185** | (0.614) | 0.561 | (0.203) | 2.009** | (0.528) |
|  |  |  |  |  |  |  |  |  |
| **Controls from 2018/19** |  |  |  |  |  |  |  |  |
| Male | 0.763 | (0.234) | 1.649* | (0.416) | 0.914 | (0.261) | 1.434 | (0.344) |
| Age | 1.034 | (0.028) | 1.020 | (0.025) | 0.991 | (0.025) | 1.018 | (0.027) |
| Non-white | 1.957 | (1.009) | 1.700 | (0.684) | 4.254** | (2.183) | 1.311 | (0.554) |
| Partnered | 2.569* | (1.005) | 0.868 | (0.245) | 1.164 | (0.376) | 1.017 | (0.300) |
| Have children in BU | 0.791 | (0.355) | 1.413 | (0.511) | 0.736 | (0.321) | 1.304 | (0.410) |
| Live in rural area | 0.631 | (0.230) | 0.901 | (0.259) | 0.439* | (0.159) | 1.610 | (0.417) |
| Limiting, long-term illness | 0.909 | (0.381) | 0.627 | (0.251) | 1.049 | (0.451) | 0.526 | (0.198) |
| Depressive symptomology | 1.245 | (0.731) | 1.094 | (0.486) | 0.271 | (0.240) | 1.940 | (0.794) |
| Has degree [NVQ4-5] | 0.930 | (0.292) | 1.491 | (0.408) | 0.623 | (0.206) | 1.274 | (0.353) |
| Social class |  |  |  |  |  |  |  |  |
| Manag, admin, prof. |  |  |  |  |  |  |  |  |
| Intermediate | 0.976 | (0.499) | 0.900 | (0.369) | 0.591 | (0.289) | 0.690 | (0.272) |
| Routine/manual | 0.699 | (0.317) | 1.006 | (0.407) | 0.954 | (0.403) | 1.194 | (0.423) |
| Other/incomplete info | 1.267 | (0.467) | 1.214 | (0.445) | 0.701 | (0.267) | 1.098 | (0.348) |
| Index of Multiple Dep. |  |  |  |  |  |  |  |  |
| Quintile 1 (least dep.) |  |  |  |  |  |  |  |  |
| Quintile 2 | 0.790 | (0.282) | 1.009 | (0.367) | 1.157 | (0.432) | 1.080 | (0.334) |
| Quintile 3 | 0.290** | (0.134) | 1.061 | (0.436) | 0.698 | (0.291) | 0.784 | (0.267) |
| Quintile 4 | 0.481 | (0.205) | 1.886 | (0.742) | 0.502 | (0.209) | 1.081 | (0.412) |
| Quintile 5 (most dep.) | 0.404 | (0.259) | 0.421 | (0.226) | 0.642 | (0.353) | 0.741 | (0.399) |
| Financial difficulties | 0.911 | (0.597) | 0.923 | (0.368) | 0.264 | (0.228) | 0.508 | (0.195) |
| Own home | 1.965* | (0.613) | 1.268 | (0.366) | 1.845 | (0.591) | 0.778 | (0.207) |
| Log wealth | 0.993 | (0.044) | 1.011 | (0.025) | 1.029 | (0.068) | 0.997 | (0.026) |
|  |  |  |  |  |  |  |  |  |
| **Controls from 2020** |  |  |  |  |  |  |  |  |
| Have private pension | 2.181* | (0.706) | 1.489 | (0.412) | 1.272 | (0.359) | 1.298 | (0.325) |
| Currently working | 1.557 | (0.806) | 0.829 | (0.296) | 0.277** | (0.119) | 1.254 | (0.532) |
| Financial condition due to COVID |  |  |  |  |  |  |  |  |
| Better off | 1.226 | (0.464) | 1.660 | (0.654) | 1.637 | (0.581) | 2.079* | (0.656) |
| Same (ref.) |  |  |  |  |  |  |  |  |
| Worse off | 2.048* | (0.748) | 3.154** | (0.933) | 1.782 | (0.627) | 3.514** | (0.985) |
| Covid exposure | 1.186 | (0.396) | 1.045 | (0.324) | 1.088 | (0.292) | 0.858 | (0.214) |
| *N* | 1,354 |  |  |  | 1,201 |  |  |  |

Notes: Figures are relative-risk ratios. **p*<0.05, ***p*<0.01.

**Table A4.** Multinomial logistic regressions with interactions using inverse probability weighting

|  | Covid Wave 1 (Jun/Jul 2020) | | | | | | | | Covid Wave 2 (Nov/Dec 2020) | | | | | | | |
| --- | --- | --- | --- | --- | --- | --- | --- | --- | --- | --- | --- | --- | --- | --- | --- | --- |
| Ref: no change | Retiring earlier | | | | Retiring later | | | | Retiring earlier | | | | Retiring later | | | |
| (A) Self-rated health |  |  |  |  |  |  |  |  |  |  |  |  |  |  |  |  |
| Financial insecurity | 0.808 | (0.272) | 0.760 | (0.271) | 2.185** | (0.614) | 1.661 | (0.541) | 0.561 | (0.203) | 0.557 | (0.217) | 2.009** | (0.528) | 2.029** | (0.539) |
| Poor health | 1.044 | (0.505) | 0.854 | (0.497) | 1.898* | (0.597) | 0.411 | (0.259) | 0.626 | (0.278) | 0.616 | (0.312) | 0.487* | (0.175) | 0.525 | (0.335) |
| Interaction terms between insecurity and poor health |  |  | 1.522 | (1.348) |  |  | 6.995** | (4.744) |  |  | 1.044 | (0.912) |  |  | 0.907 | (0.659) |
|  |  |  |  |  |  |  |  |  |  |  |  |  |  |  |  |  |
| (B) Depressive symp. |  |  |  |  |  |  |  |  |  |  |  |  |  |  |  |  |
| Financial insecurity | 0.808 | (0.272) | 0.937 | (0.343) | 2.185** | (0.614) | 1.869* | (0.579) | 0.561 | (0.203) | 0.618 | (0.263) | 2.009** | (0.528) | 1.774 | (0.522) |
| Depressive symp. | 0.865 | (0.375) | 1.217 | (0.638) | 1.348 | (0.427) | 0.639 | (0.480) | 1.438 | (0.610) | 1.611 | (0.740) | 1.866* | (0.481) | 1.369 | (0.658) |
| Interaction terms between insecurity and depressive symp. |  |  | 0.438 | (0.384) |  |  | 2.671 | (2.446) |  |  | 0.745 | (0.541) |  |  | 1.577 | (0.902) |

Note: All panels represent different regressions. Each regression includes the same set of controls and modifiers as in Table 1. **p*<0.05, ***p*<0.01.

**Table A5.** Panel multinomial logistic regressions with interactions (random effects model)

| Ref: no change | Retiring earlier | | Retiring later | |
| --- | --- | --- | --- | --- |
| (A) Self-rated health |  |  |  |  |
| Financial insecurity | 0.578 | (0.214) | 2.938** | (1.067) |
| Poor health | 0.514 | (0.317) | 0.459 | (0.365) |
| Insecure * poor health | 1.580 | (1.377) | 2.728 | (2.436) |
|  |  |  |  |  |
| (B) Depressive symp. |  |  |  |  |
| Financial insecurity | 0.796 | (0.293) | 2.700** | (1.014) |
| Depressed | 1.769 | (0.834) | 0.847 | (0.547) |
| Insecure * depressed | 0.361 | (0.238) | 2.620 | (1.976) |

Note: All panels represent different regressions. Each regression includes the same set of controls and modifiers as in Table 3. **p*<0.05, ***p*<0.01.
